# Complete pathological response of post neoadjuvant chemotherapy in colorectal cancer with liver metastasis: a case report

**DOI:** 10.1093/jscr/rjac041

**Published:** 2022-02-19

**Authors:** Ragad I Al Jazzar, Mohammed Algarni, Sarah Al-Maiman, Nayef Alzahrani

**Affiliations:** 1 College of Medicine, King Saud bin Abdulaziz University for Health Sciences, Riyadh, Saudi Arabia; 2 King Saud bin Abdulaziz University for Health Sciences, Oncology Department, Ministry of National Guard—Health Affairs, Saudi Arabia, King Abdullah International Medical Research Center, Riyadh, Saudi Arabia; 3 Consultant Anatomic Pathology, GI, Pancreatobillary and Liver Pathology, King Abdulaziz Medical City, Ministry of National Guard Health Affairs, Riyadh, Saudi Arabia; 4 Department of Surgery, King Abdulaziz Medical City, Ministry of National Guard Health Affairs, Riyadh, Saudi Arabia

## Abstract

Treatment of metastatic colorectal cancer has evolved throughout the years and various methods have been proposed to reach a pathological complete response state. We report a case of a 73-year-old male presented with a sigmoid adenocarcinoma with two synchronous liver metastases. The patient received five cycles of FOLFOX neoadjuvant chemotherapy, 41% reduction of tumor size was noted upon reassessment. Therefore, a low anterior resection of the rectum and synchronous resection of segment 5 and 8 of the liver was done along with right-sided diaphragmatic stripping. A pathological complete response was achieved in both primary and secondary tumors that are considered rare and challenging in metastatic colorectal cancer**.** Neoadjuvant chemotherapy showed promising findings in advanced colorectal cancer.

## INTRODUCTION

Colorectal cancer (CRC) is considered the third most common type of malignancy worldwide and the second leading cause of death in 2020 according to the WHO [1]. The Saudi cancer registry revealed that 1659 cases of CRC were diagnosed in 2016. Male gender was predominant with a ratio of 136:100 male to female, moreover, making CRC the first common cancer in males and the third among females [2]. Approximately 20–35% of patients develop liver metastasis during the course of the disease [3]. Synchronous liver metastasis with CRC was identified in 15–25% of patients at the time of presentation [4]. The management of CRC with liver metastasis is multimodal consisting of chemotherapy, radiotherapy and surgery [5]. The mainstay treatment that is associated with a 5-year survival up to 58% in colorectal liver metastasis was found to be in liver resection [6]. Unfortunately not all patients are amenable to undergo liver resection. Therefore, neoadjuvant chemotherapy (NAC) qualifies them for secondary resection after achieving a reduction in tumor size and controlling the micrometastasis. Pathological complete response (pCR), which implies the absence of all residual invasive cancer cells, was recognized after NAC and was associated with a 10-year survival of 85.2% and a disease free state of patients 73.7% whom are known to have liver metastasis [7].

We report a case of pCR after five cycles of FOLFOX consisting of a combination of leucovorin, 5-fluorouracil and Oxaplatin.

## CASE REPORT

**Figure 1:**
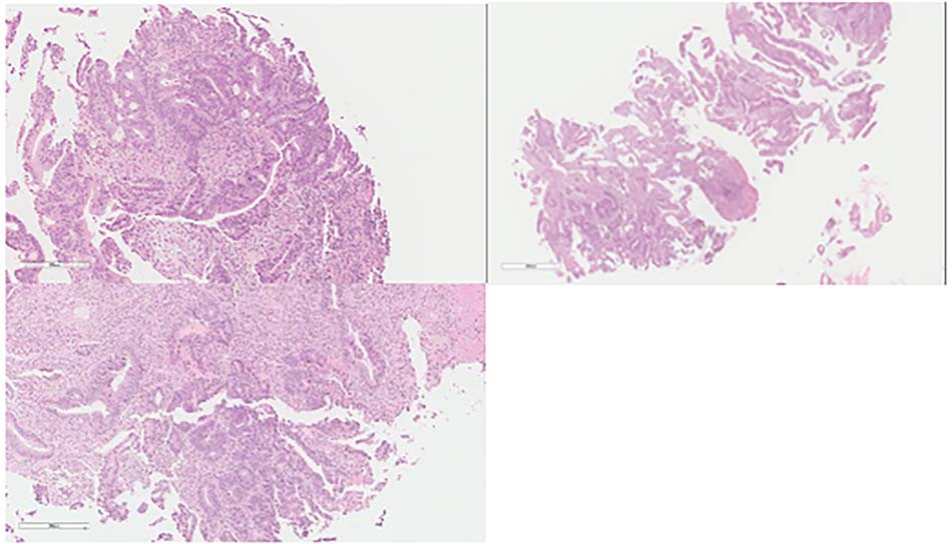
Colon: status of pre NAC: colonic mucosa with an infiltrative malignancy formed by tubules and gland of epithelial cells with high nucleus to cytoplasm ratio. The cells are large with frequent mitosis.

**Figure 2:**
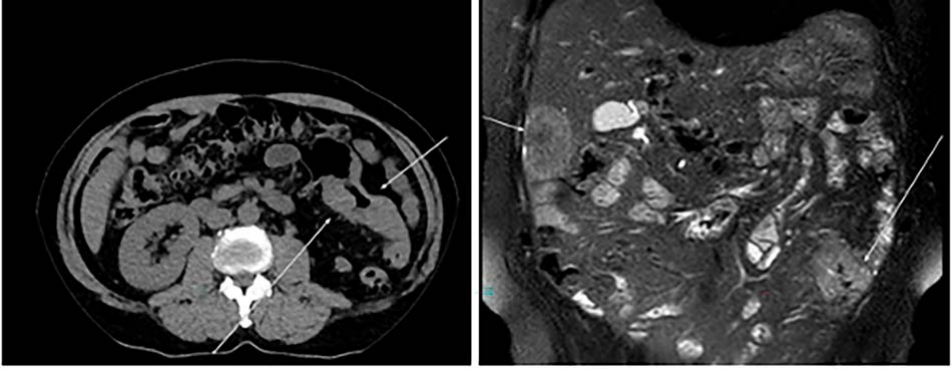
CT: status of pre administration of NAC.

**Figure 3:**
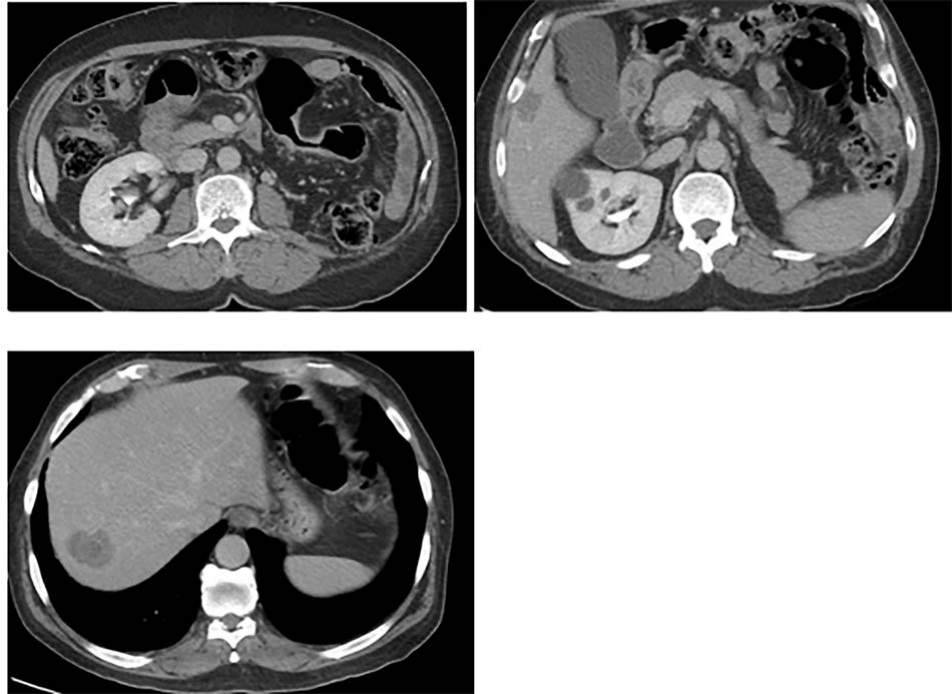
CT: status of post administration of NAC.

An otherwise healthy 73-year-old male, presented with rectal bleeding 6 months back. Colonoscopy revealed a sigmoid colon mass about 25 cm from the anal verge extending 10 cm proximally and a biopsy confirmed invasive adenocarcinoma (Fig. 1) Computed tomography of the abdomen and pelvis revealed a circumferential wall thickening involving the distal colon/proximal sigmoid colon spanning for 6 cm with maximum thickness of 2.3 cm, and it was also associated with surrounding fat stranding and multiple regional lymph nodes. Liver lesions were described as ill-defined hypodense lesions in two segments. The first lesion was seen in segment 7 measuring 6 × 7.5 cm and the other was seen in segment 5 measuring 6 × 3 cm (Fig. 2). Furthermore, chest CT showed no lung metastasis and a carcinoembryonic antigen level of 24.8. Eventually, a diagnosis of metastatic sigmoid adenocarcinoma with liver lesions was made. The multidisciplinary team decided to start the patient on Nac and then the case was reassessed for further resection. After completing five cycles FOLFOX, a CT of chest, abdomen and pelvis was performed for evaluating the response. The primary descending colon tumor demonstrated reduction in bulk with persistent serosal irregularity and no definitive regional lymphadenopathy was detected. The hepatic lesions revealed a partial response; in segment 7, the lesion is currently measuring 3.9 cm and in segment 5, the lesion measures 2.7 cm. Partial response to therapy was noted by a 41% reduction in sum tumor burden as per RECIST criteria (Fig. 3). The clinical tumor node metastasis classification post chemo was calculated to be T2N0M1 for descending colon cancer along with a CEA of <1.7. Despite not adding a biological agent with the systemic therapy which is the standard protocol in metastatic CRC, the patient achieved a remarkable reduction in tumor burden. The patient underwent a laparotomy with a low anterior resection of the rectum and anastomosis, synchronous resection of segments 6 and 8 of the liver along with a right-sided diaphragmatic stripping.

**Figure 4:**
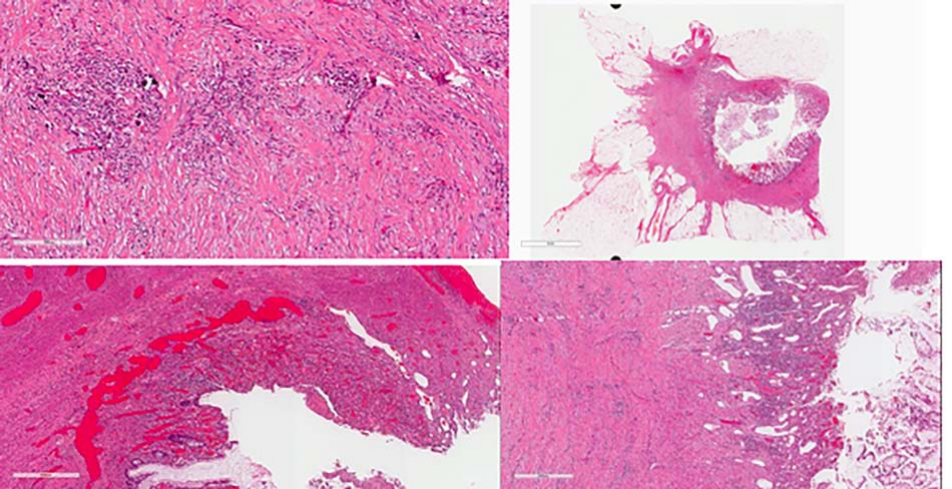
Colon: status of post NAC with complete response. Mucosal ulcer, fibrosis, calcification and histolytic collection with no tumor cells.

The patient was clinically and vitally stable post operation and was recovering well upon discharge. Pathology revealed no residual tumor, extensive necrosis and calcification with no viable cancer cells with a complete response and a score of 0. Moreover, the sigmoid colon specimen revealed no viable cancer cells, complete response, a score of 0 and nine lymph nodes were found to be negative to malignancy (0/9) (Fig. 4).

## DISCUSSION

The management of stage IV CRC can be either curative or palliative depending on the resectability of the metastatic disease**.** Excising the primary lesion is considered the cornerstone treatment for resectable tumors. There are different approaches to the surgery in patients with CRC with synchronous liver metastasis: liver first, bowel first and simultaneous resection of both primary and metastatic lesions. Raphael et al. meta-analysis compared the findings of previous studies with a goal of knowing the long-term outcome of chemotherapy in CRC with resectable liver metastases, and patients were subdivided into two groups, surgery alone and surgery with the administration of systemic chemotherapy regardless of its setting. Three of the included studies that are within the systemic chemotherapy group received six cycles of FOLFOX. The findings demonstrated an improvement in the overall survival rates for 23% of patients in the surgery and chemotherapy group compared with surgery alone. Twenty-nine percent of whom showed an improvement in relapse free survival were found to be in chemotherapy group in comparison to the surgery alone group [6]. Palliative treatment is recommended for a subset of patients with unresectable metastatic tumors who are not candidates for surgery [8].

Preoperative administration of NAC helps in reducing the tumor size and offers resectability with a curative intent. Disappearance of tumors in imaging post chemotherapy cycles does not always correlate with a pCR state, and may lead to relapses if left unresected. According to Adam et al., a pCR is an extremely rare finding and accounts for 4% of patients post treatment of metastatic CRC with preoperative chemotherapy. Furthermore, pCR carries a good prognosis with a 5-year survival reaching 76% [9].

A pCR was noted in a previous case report after seven cycles of CAPOX and bevacizumab, an exceptional response with 70% regression of metastatic tumor along with a complete regression of primary tumor [10]. Bulajic et al’s are the only study in literature that used chemotherapy in a neoadjuvant setting and then proceeded to resect primary and secondary tumors simultaneously [10]. Other authors reported similar findings but with a different approach by resecting the primary tumor, then administering NAC and lastly resecting the secondary lesions. A liver-first approach was also stated in literature with a result of pCR [11].

The survival rate in CRC with a single organ metastasis is higher due to the curative resection that could be performed in combination with systemic therapy in which they are candidates for in comparison to multi-organ metastases [4]. Curative therapy was seen in patients with resectable liver metastasis along with chemotherapy with a 5-year overall survival rate up to 50% [12]. Lydia et al. reported a higher survival rate in patients who underwent metastasectomies with or without other treatments from a median of 25 months to 46.2 months. In patients who received systemic chemotherapy, the survival rate increased from 12.1 months to 15.3 months without undergoing metastasectomies [4]. Metastatic CRC necessitates a well-tailored treatment plan to improve the patient’s quality of life and increase the disease free state. This report aims to shed the light of such rare cases in literature.
